# OXA-900, a Novel OXA Sub-Family Carbapenemase Identified in *Citrobacter freundii*, Evades Detection by Commercial Molecular Diagnostics Tests

**DOI:** 10.3390/microorganisms9091898

**Published:** 2021-09-07

**Authors:** Sammy Frenk, Nadya Rakovitsky, Hadas Kon, Reut Rov, Shirin Abramov, Mor Nadia Lurie-Weinberger, David Schwartz, Erica Pinco, Jonathan Lellouche, Yehuda Carmeli

**Affiliations:** 1 National Institute for Antibiotic Resistance and Infection Control, Ministry of Health, Tel Aviv 6423906, Israel; sammyfrenk@gmail.com (S.F.); nadyarak@tlvmc.gov.il (N.R.); hadaskon@tlvmc.gov.il (H.K.); reutrov@tlvmc.gov.il (R.R.); shirinab@tlvmc.gov.il (S.A.); morlw@tlvmc.gov.il (M.N.L.-W.); davidsc@tlvmc.gov.il (D.S.); jonathanl@tlvmc.gov.il (J.L.); 2 Shmuel Harofeh Geriatric Medical Center, Be’er Ya’akov 70350, Israel; erica.pinco@MOH.GOV.IL; 3 The Miriam and Sheldon Adelson School of Medicine, Ariel University, Ariel 40700, Israel; 4 Sackler Faculty of Medicine, Tel Aviv University, Tel Aviv 6997801, Israel

**Keywords:** carbapenemase, β-lactamase, *bla*_OXA-48-like_, Enterobacterales

## Abstract

Using whole-genome sequencing and cloning of the target gene, we identified *bla*_OXA-900_ carbapenemase, a novel *bla*_OXA_ belonging to a distant and distinct sub-family of *bla*_OXA-48-like_. The plasmid-mediated gene was identified in a *C. freundii* isolate with elevated carbapenem MICs that evaded detection by commercial DNA-based methods. The novel gene, an OXA-48 family carbapenem-hydrolyzing class D β-lactamase, OXA-900, likely originates from marine environmental *Shewanella*. Since this plasmid-mediated gene has entered a member of the Enterobacterales and evades detection by commonly used tests, it may gain wide dissemination among Enterobacterales.

## 1. Introduction

Detection of organisms carrying carbapenemases is important for infection control and clinical decision making. Commercial molecular tests target specific, known carbapenemases. Thus, novel carbapenemases may evade detection. OXA-48 family carbapenem-hydrolyzing [Ambler] class D β-lactamases (CHDL) in Enterobacterales are a common enzyme group that was first reported in 2004 in *Klebsiella pneumoniae* isolated from a patient in Turkey [1]. *bla*_OXA-48-like_ genes are found mainly in *K. pneumoniae* but also in other pathogenic Enterobacterales [2]. The hydrolytic activity of OXA-48-like CHDLs against carbapenems is relatively low and only moderately increases MIC values when it is the sole resistance mechanism [2]. However, high MICs for carbapenems are reported in Enterobacterales when OXA-48-like enzymes are produced in strains lacking OmpF and/or OmpC type porins [3]. The latest version (Nov. 2019) of the NCBI Bacterial Antimicrobial Resistance Reference Gene Database held about 40 variants of *bla*_OXA-48-like_ genes. As *bla*_OXA-48-like_ genes are genetically conserved, DNA-based tests for their detection are reliable. In clinical settings, most tests for the detection of *bla*_OXA-48-like_/OXA-48-like are DNA based, focusing mainly on the *bla*_OXA-48_ family variants including *bla*_OXA-181_ sub-type but not *bla*_OXA-54_. Here, we describe a novel *bla*_OXA_ belonging to a distant and distinct sub-family of *bla*_OXA-48-like_ genes identified in a *C. freundii* isolate with elevated carbapenem MICs, which evaded detection by DNA-based methods.

## 2. Materials and Methods

### 2.1. Description of the Isolate

The specimen was a rectal swab obtained for routine surveillance of carbapenemase-producing Enterobacterales (CPEs) in an Israeli long-term care facility in 2019. A suspected CRE colony was identified by visual inspection on selective chromogenic media (CHROMagar^TM^ mSuperCarba^TM^, HyLabs, Rehovot, Israel). The isolate was identified by Vitek 2^™^ (bioMérieux, Marcy-l′Étoile, France) as *C. freundii* with intermediate resistance to ertapenem (1 µg/mL). Tests for the presence of common carbapenemase genes using Xpert^®^ Carba-R assay on a GeneXpert^®^ system (Cepheid, Sunnyvale, CA, USA) were negative. The specimen was named isolate ISCF142 and sent to the National Laboratory for Antibiotic Resistance for further evaluation.

### 2.2. Antibiotic Susceptibly Testing

Antibiotic susceptibility testing was performed using broth microdilution (Sensititre™ GN6F plate, ThermoFisher Scientific, Oakwood Village, OH, USA) according to CLSI M07 guidelines and manufacturer’s instructions. For isolates with carbapenem MIC values below the detection level of the Sensititre™ GN6F plate, homemade assays containing ertapenem, imipenem, and meropenem were used. These were produced according to CLSI guidelines using recommended control strains [4]. Susceptibility was determined using CLSI 2019 breakpoints.

### 2.3. Detection of Carbapenemases

Carbapenemase activity was determined by the β-CARBA^™^ assay (Bio-Rad Laboratories, Hercules, CA, USA) as well as by a modified carbapenem inactivation method [5].

Three commercial tests and a homemade method were used to detect carbapenemase genes: (1) Xpert^®^ Carba-R assay, (2) Pneumonia *plus* Panel assay on a BIOFIRE^®^ FILMARRAY^®^ system (bioMérieux), and (3) CarbaR^+^ panel on a Novodiag^®^ device (Mobidiag, Keilaranta, Finland). These tests detect *bla*_KPC_, *bla*_NDM_, *bla*_VIM_, *bla*_IMP-1_ and *bla*_OXA-48/181_. We performed a homemade PCR that detects these carbapenemases as described previously [6] and also *bla*_IMI_ using the IMI-F (5′-GCCATATCACCTAATGACATTCC-3′) and IMI-R (5′-GCAAATGAACGATTTCCATTATGTA-3′) primers.

We used the NG-test CARBA 5 (NG Biotech, Guipry, France) immunochromatographic assay, a rapid multiplex lateral flow assay, for phenotypic detection and differentiation of KPC, OXA-48-like, VIM, IMP, and NDM carbapenemases according to the manufacturer’s instructions.

### 2.4. WGS Analysis

DNA was extracted using the MagAttract HMW DNA Kit (Qiagen, Hilden, Germany) and sequenced at the Sequencing Core at the University of Illinois at Chicago according to their standard protocol for NextSeq500 (Illumina Inc., San Diego, CA, USA). Long-read sequencing was carried out using the SQK-RBK004 kit (Oxford Nanopore Technologies (ONT), Oxford, UK) on the MinION (ONT) with Guppy software (ONT) according to the manufacturer’s instructions. Illumina reads were quality screened using Fastp [7], assembled with long reads using Unicycler [8], and annotated using Prokka [9]. Strain type was predicted using pubMLST’s *C. freundii* scheme (https://pubmlst.org/cfreundii/, accessed on 1 August 2020). ORFs were searched against the CARD and NCBI databases using DIAMOND BLAST [10]. Whole-genome sequencing (WGS) using both short-read and long-read technologies was used as previously described [11].

### 2.5. Insertion Sequences (IS)

IS were identified by Prokka annotation, and the inverted repeat sequences, left and right (IRL and IRR, respectively), were identified using the ISfinder database [12]. Their location was plotted manually on a genomic illustration produced by EasyFig [13].

### 2.6. Carbapenemase Gene Cloning and Functional Confirmation

Cloning was performed by amplifying the suspected carbapenemase gene using a primer set targeting the first and last 20 bases of the gene with added restriction sites XbaI and EcoRI. The resulting fragment was cleaned using the commercial kit Nucleospin gel and PCR cleanup (MACHEREY-NAGEL GmbH & Co, Düren, Germany). The clean fragment was subjected to cutting by XbaI and EcoRI restriction enzyme (New England Biolabs, Ipswich, MA, USA) and cleanup, as stated above. The vector pHSG396 (Takara Bio, Saint-Germain-en-Laye, France) was subjected to the same restriction enzymes and separated by agarose gel electrophoresis, followed by Nucleospin gel and PCR cleanup kit. The vector and insert were ligated by T4 DNA Ligase (New England Biolabs). The circular plasmid with the gene was inserted into MAX Efficiency™ DH10β Competent Cells (Invitrogen, Waltham, MA, USA). The resulting plasmids were extracted using NucleoSpin^®^ Plasmid EasyPure (MACHEREY-NAGEL). The success of cloning was evaluated by positive PCR for the target gene. The modified carbapenem inactivation method was used on the *C. freundii* isolate, the transformant *E. coli* DH10β with the suspected carbapenemase ORF, and *E. coli* DH10β with the pHSG396 plasmid but no insert (as a negative control).

### 2.7. Phylogenetic Analysis of Selected bla_OXA_ Genes

The suspected *bla*_OXA_ gene was compared to the known alleles found at the NCBI Bacterial Antimicrobial Resistance Reference Gene Database (PRJNA313047). All sequences were aligned using MAFFT software [14]. The resulting alignment (only of genes diversified from *bla*_OXA-55_) was interpreted by constructing a maximum likelihood phylogenetic tree with 100 permutations using RAxML [15]. The resulting analysis was visualized using Dendroscope software [16].

### 2.8. Conjugation Efficiency

*E. coli* DH10β with and without a chloramphenicol resistance gene was used as the recipient for mating experiments; the donor strain was *C. freundii* ISF142. These experiments included solid mating (0.45 µm pore-size nitrocellulose MF membrane filters, Merck Millipore Ltd., Cork, Ireland) placed on 5% blood agar plates (Hylabs)) and liquid mating (mixed culture left overnight at 35 °C) with both selective media (ertapenem 0.5 µg/mL and 25 µg/mL chloramphenicol) and selective chromogenic media (CHROMagar^TM^ mSuperCarba^TM^, HyLabs, Rehovot, Israel) for resistant recipient screening.

### 2.9. Data Availability

Data were submitted under NCBI BioSample Accession Number SAMN13412315 and Assembly Numbers CP046502-CP046506. The new *bla*_OXA_ was named *bla*_OXA-900_ and can be found under the accession number MN936180.

## 3. Results

The three commonly used commercial molecular tests targeting *bla*_KPC_, *bla*_NDM_, *bla*_VIM_, *bla*_IMP-1,_ and *bla*_OXA-48/181_ failed to detect a carbapenemase, as did an in-house PCR method. However, two carbapenem hydrolysis tests, the β-CARBA^™^ assay and the modified carbapenem inactivation method, confirmed carbapenemase activity. The lateral flow assay confirmed the presence of an OXA-48 type carbapenemase.

WGS enabled the assembly of a circular chromosome and four plasmids (NCBI BioSample Accession Number SAMN13412315 and Assembly Numbers CP046502-CP046506). The isolate was identified as *C. freundii*, ST111. ISCF142 carried two chromosomal antibiotic resistance genes (ARGs), *qnrB*38 and *ampC* (*bla*_CMY-65_), and several plasmid-coded ARGs (Table A1). Among these ARGs were the β-lactamase genes *bla*_CTX-M-3_, *bla*_CTX-M-39_, and *bla*_PER-2_, which may contribute to *C. freundii* ISCF142’s resistance to third-generation cephalosporins and aztreonam (Table 1). A fifth gene was also found, encoding a novel putative β-lactamase first annotated as a *bla*_OXA-54_ distant variant: the gene *bla*_OXA-900_.

We cloned *bla*_OXA-900_ into an *E. coli* DH10β in order to evaluate its activity toward several antibiotics by broth microdilution. Results of antibiotic susceptibility testing are shown in Table 1. The resulting transformant was susceptible to commonly used cephalosporins, intermediate to ertapenem (1 µg/mL), and had increased MICs to meropenem (1 µg/mL) and imipenem (2 µg/mL), compared to the susceptible *E. coli* DH10β.

**Figure 1:**
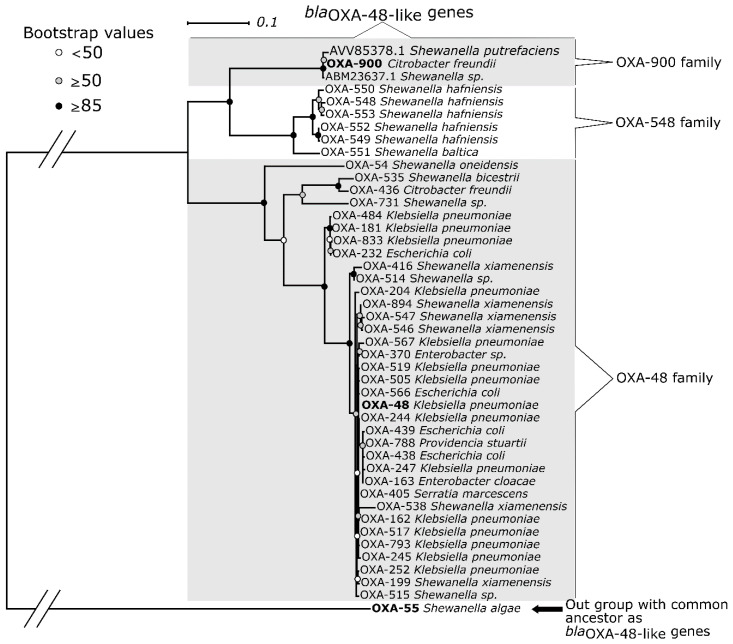
Maximum Likelihood phylogenetic tree analysis of all *bla*_OXA_ that diversify from *bla*_OXA-55_ found at the NCBI’s Bacterial Antimicrobial Resistance Reference Gene Database. Two sequences from environmentally isolated *Shewanella* were also included, the first from *S. putrefaciens* isolated from *Litopenaeus vannamei* (whiteleg shrimp) in China (AVV85378.1) and the second (ABM23637.1) from *Shewanella* sp. from the marine environment.

Comparing *bla*_OXA-900_ to other *bla*_OXA-48-like_ genes in the NCBI Bacterial ARG database, the two best hits were from environmentally isolated organisms (Figure 1). *bla*_OXA-900_ was most similar (99% and 98% similarity) to two genes found previously in *Shewanella putrefaciens*, a saprophytic warm-climate marine organism, genomes that were not formally reported or characterized. *bla*_OXA-900_ also had high deduced amino acid similarity to *bla*_OXA-548_ (83.5%), *bla*_OXA-48_ (81.1%), and *bla*_OXA-181_ (80.7%).

**Figure 2:**
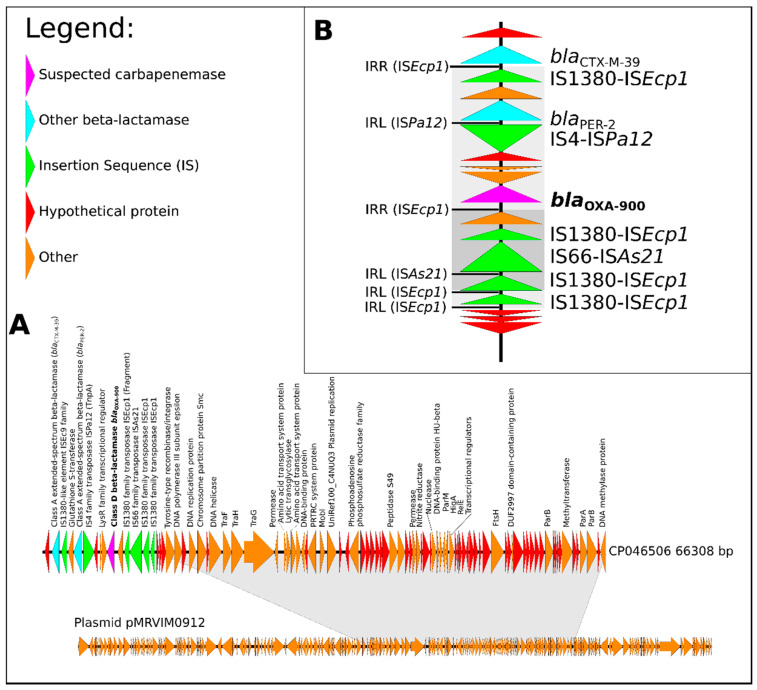
Genetic analysis of *C. freundii* plasmid CP046506 and its antibiotic resistance islands (ARI): (**A**) plasmid CP046506, a version of pMRVIM0912 with an ARI on which the new *bla*_OXA-900_ was found (regions of similarity are marked in grey); (**B**) ARI with two ESBL genes and a novel carbapenemase. All IS inverted repeats on the right (IRR) and on the left (IRL) are marked and the region suggested to be inserted by the IS*Ecp1*is marked in grey shades. Triangles represent ORFs: hypothetical proteins are marked in red, annotated genes in orange, IS fragments in green, the carbapenemase is marked in purple and other β-lactamases in blue.

The *bla*_OXA-900_ gene was located on an antibiotic resistance island (ARI) on an IncC plasmid (CP046506). Plasmid CP046506 lacks substantial parts compared to a previously reported *C. freundii* IncC plasmid—namely, pMRVIM0912 [17] (Figure 2a). CP046506 includes replication, conjugation, and addiction elements characteristic of IncC. Mating experiments did not yield any resistant recipient cells, suggesting low conjugative potential with *E. coli*. Further analysis showed that this plasmid contains six genes previously shown to play a role in conjugation [18]: *traFHG, mobI,* and the regulatory gene *acaCD.* Notably, the gene *traI* was not found in CP046506, nor were other *tra* genes that were predicted to play a part in conjugation in silico [18], indicating a generally reduced potential for conjugation.

We analyzed the mechanisms by which OXA-900 entered Enterobacerales. The ARI and the IS involved are grouped by their location on the plasmid backbone [17]. In this study, the CP046506 plasmid includes an ARI with an IS*Ecp1* insertion sequence carrying a bla_CTX-M-39_ that was previously reported on type 2 IncC plasmids in a location different from IncC ARI types A and B—namely, RI-5 [17]. The resulting plasmid with its uncommon resistance genes likely reflects recombination next to a RecHS recombination hotspot [17]. The location of the inverted repeats suggests that the fragment carrying the *bla*_OXA-900_ gene and several other genes (including *bla*_PER-2_) was inserted by the IS*1380* family transposase IS*Ecp1*, as it was the sole IS found enclosing the fragment by inverted repeat sequences on the right (IRR) and on the left (IRL) (Figure 2b). This IS, similar to other known mobile elements, has an important role in the evolution of multi-resistant plasmids [19,20].

## 4. Discussion

Here, we report on a novel OXA-48-like carbapenemase named OXA-900. This enzyme likely originated from marine environmental *Shewanella*. It avoids detection by commercially available DNA-based tests but is detectable by a lateral flow protein-based assay. The OXA-900 has carbapenemase activity, leading in transformants to increased carbapenem MICs in the range reported for other CHDLs [2]. OXA-900 belongs to a distant family related to OXA-48 that until now has not been described in human pathogens.

Antibiotic resistance genes that originate from environmental organisms are an important source of resistance in human pathogens. For example, *bla*_CTX-m_ genes likely emerged from *Kluyvera* [21], *bla*_NDM_ has origins in *Erythrobacter litoralis* [22], and *qnrA* is found in *Shewanella algae* [23]. Moreover, the *bla*_OXA-48-like_ genes that gained wide distribution in human pathogens originate from *Shewanella* spp. [24,25]. Therefore, OXA-900’s introduction from an environmental organism into Enterobacterales poses the imminent threat that it may further spread and establish a reservoir in human pathogens. Since it eludes detection by the common commercial diagnostic methods, it may escape infection control efforts to limit the spread of carbapenemases.

*bla*_OXA-900_ represents a distinct family of OXA enzymes. In their overview of *Shewanella bla*_OXA-48-like_ genes, Tacão et al. [25] presented this genus’s diversity of *bla*_OXA-48-like_ genes in the environment. One can group these enzymes into three major clusters: the *bla*_OXA-48_ family, the *bla*_OXA-548_ family, and a third cluster that includes relatives of the *bla*_OXA-900_ described here (named the *bla*_OXA-900_ family).

We conclude that OXA-900 has the potential to become a widespread resistance determinant in Enterobacterales. Diagnostic tests should be adapted to detect the *bla*_OXA-900_ family.

## Figures and Tables

**Table 1:** Antibiotic susceptibility profile by broth microdilution of *C. freundii* ISCF142, *E. coli* DH10β-harboring recombinant β-lactamase *bla*_OXA-900_, and *E. coli* DH10β without the β-lactamase gene insert (negative control).

		MIC in µg/mL (CLSI Breakpoints)		
Antibiotic Class	Antibiotic	*C. freundii* Isolate ISCF142	Transformant DH10β (OXA-900)	DH10β (Negative Control)
Carbapenems	Meropenem	1 (S)	1 (S)	<0.12 (S)
	Imipenem	1 (S)	2 (S)	<0.5 (S)
	Ertapenem	1 (I)	1 (I)	<0.12 (S)
Cephalosporins	Cefotaxime	8 (R)	<0.5 (S)	<0.5 (S)
	Ceftazidime	>16 (R)	<0.5 (S)	<0.5 (S)
Cephalosporin combinations	Ceftazidime/ avibactam ^a^	<0.5 (S)	<0.5 (S)	<0.5 (S)
	Ceftolozane/ tazobactam ^a^	>32 (R)	8 (S)	<0.5 (S)
Monobactams	Aztreonam	>32 (R)	<0.5 (S)	<0.5 (S)
Penicillin combinations	Piperacillin/ tazobactam ^a^	>32 (R)	>32 (R)	2 (S)
	Amoxicillin/clavulanic acid ^b^	>64 (R)	>64 (R)	<4 (S)
Fluoroquinolones	Ciprofloxacin	0.25 (S)	0.12 (S)	0.12 (S)
Polymixins	Colistin	1	0.25	0.5
Aminoglycosides	Tobramycin	1 (S)	<1 (S)	<1 (S)
Tetracyclines	Tigecycline	0.25 (S)	<0.25 (S)	<0.25 (S)
Sulfonamide combinations	Trimethoprim/ sulfamethoxazole ^c^	1 (S)	<1 (S)	<1 (S)

^a^ Tazobactam and avibactam at a fixed concentration of 4 µg/mL. ^b^ Clavulanic acid at a fixed concentration of 2 µg/mL. ^c^ Sulfamethoxazole at a fixed concentration of 19 µg/mL.

## Data Availability

Data were submitted under the NCBI BioSample accession number SAMN13412315 and assembly numbers CP046502-CP046506. The new *bla*_OXA_ was named *bla*_OXA-900_ and can be found under the accession number MN936180.

## Appendix A

**Table A1:** Resistance genes found on genomic fragments assembled from *C. freundii* ISCF142.

Resistance Mechanism	Antibiotic Resistance Gene	Best Hit Identity (%)	Query/Template Length	Genetic Fragment
Quinolone resistance protein Class C β-lactamases	*qnrB*38-like	99.53	645/645	Chromosome (CP046502)
	*bla*_CMY-65-like_	99.91	1146/1146	
Efflux pumps	*tet*(D)	100	1185/1185	Plasmid (CP046504)
Class A β-lactamases	*bla*_CTX-M-3_	100	876/876	
Dihydropteroate synthase	*sul*2	100	816/816	
Class A β-lactamases	*bla*_CTX-M-39_	100	876/876	Plasmid (CP046506)
Class A β-lactamases	*bla_PER-2_*	100	927/927	
Class D β-lactamases	*bla_OXA-900_*	81.5 *	810/795	

* with bla_OXA-54_.
